# Efficiency of computerized adaptive testing with a cognitively designed item bank

**DOI:** 10.3389/fpsyg.2024.1353419

**Published:** 2024-06-26

**Authors:** Hao Luo, Xiangdong Yang

**Affiliations:** Department of Educational Psychology, Faculty of Education, East China Normal University, Shanghai, China

**Keywords:** computerized adaptive testing, cognitive design system approach, item bank, item generation, linear logistic trait model

## Abstract

An item bank is key to applying computerized adaptive testing (CAT). The traditional approach to developing an item bank requires content experts to design each item individually, which is a time-consuming and costly process. The cognitive design system (CDS) approach offers a solution by automating item generation. However, the CDS approach has a specific way of calibrating or predicting item difficulty that affects the measurement efficiency of CAT. A simulation study was conducted to compare the efficiency of CAT using both calibration and prediction models. The results show that, although the predictive model (linear logistic trait model; LLTM) shows a higher root mean square error (RMSE) than the baseline model (Rasch), it requires only a few additional items to achieve comparable RMSE. Importantly, the number of additional items needed decreases as the explanatory rate of the model increases. These results indicate that the slight reduction in measurement efficiency due to prediction item difficulty is acceptable. Moreover, the use of prediction item difficulty can significantly reduce or even eliminate the need for item pretesting, thereby reducing the costs associated with item calibration.

## 1 Introduction

An item bank is key to applying computerized adaptive testing (CAT). The traditional method of developing an item bank requires content experts to meticulously design hundreds or thousands of high-quality items, and research has shown that developing a professional item bank costs hundreds or even thousands of dollars per item (Wainer, 2002). Therefore, the traditional method of developing an item bank is time consuming and costly, which seriously restricts the application of CAT.

One hopeful alternative is algorithmic or automated item-generation techniques for item development (Luecht, 2012). Item generation may produce a large number of items efficiently and quickly because of the generation rules behind the item design. Item generation can even reduce or eliminate the need for pretesting because item parameters can be predicted based on item design parameters. Item generation was divided into strong-theory and weak-theory item generation according to whether the item generation rules strictly rely on cognitive process models. Weak-theory item generation can efficiently produce CAT items through the “replacement set procedure” (Millman and Westman, 1989), but the items generated are very similar, and this method is limited by the quality of the items in the existing test. In contrast, the Cognitive Design System (CDS) approach to strong-theory item generation not only increases the efficiency of CAT item development but also improves the construct validity of the items, which is crucial for the practical application of CAT.

However, the CDS approach has a specific way of calibrating or predicting item difficulty that affects the measurement efficiency of CAT. Specifically, the CDS approach (Embretson, 1998) constructs an item bank in CAT (cognitively designed item bank) that differs from traditional item banks. Traditional item banks usually assume that items are independent of each other and use a single-level item response theory (IRT) model (e.g., Rasch, 2PL) to estimate item parameters. Instead, based on the item generation perspective, researchers have different ways. For example, a hierarchical item family model or cognitive IRT model can be used (Embretson and Yang, 2007). Therefore, the first research question is whether the use of these models for item parameter calibration affects the recovery of theta (θ) in CAT compared to traditional IRT models. In addition, models containing item design parameters, such as the linear logistic trait model (LLTM), can be used to predict item difficulty. If the predicted difficulty is used instead of the calibrated difficulty, the uncertainty in the predicted difficulty may reduce the accuracy of the item parameters, reducing the measurement efficiency of the CAT. However, using predicted difficulty can improve the efficiency of item development, reduce costs, and even eliminate the need for item pretesting. Therefore, there is a trade-off between the efficiency of item development and the measurement accuracy of CAT. The second research question is how much uncertainty in the predicted difficulty is acceptable.

These two research questions are very necessary and realistic, facilitating the integration of the CDS approach into CAT frameworks, which ultimately leads to enhanced efficiency, improved construct validity in item bank development, and cost reduction. The article is structured to first discuss the CDS approach to developing item banks, then explore the models used for calibrating or predicting item difficulty, and finally conclude with the design and results of a Monte Carlo simulation study.

## 2 Methods

### 2.1 Developing an item bank with a cognitive design system approach

First, item design variables (construct-relevant design variables) were proposed based on the cognitive model of the measured construct at the task level. Second, these variables can be combined to form several item generation rules. Finally, algorithmic or automated generation of a large number of items is achieved by changing construct-irrelevant design variables under the same item generation rule. For a more detailed development process (see Embretson, 1998). We have also developed a mental rotation CAT item bank using the Cognitive Design System approach, which is described in the Supplementary material.

### 2.2 Models for calibrating or predicting item difficulty

Cognitive IRT models include the LLTM and random-effect LLTM (RELLTM; Janssen et al., 2004). The formula for RELLTM is


$$\begin{array}{l} P\left(X_{ij}\;=\;1|\theta_{i},q_{j},\eta \right)\;=\;\frac{\text{exp}\left(\theta_{i}\;-\;\sum_{k=0}^{K}q_{jk}\eta_{k}\;+\;e_{j}\right)}{1\;+\text{ exp}\left(\theta_{i}\;-\;\sum_{k=0}^{K}q_{jk}\eta_{k}\;+\;e_{j}\right)},\\ \;e_{j}\;\sim \;N\left(0,\sigma_{\varepsilon }^{2}\right) \end{array}$$

where ***q***_*j*_ is the loading of the *K* design variables on item *j*, ***q***_*j*_={*q*_*j*0_, *q*_*j*1_, *q*_*j*2_, …, *q*_*jK*_}, **η** is the regression coefficient of the *K* design variables on item difficulty, **η**={η_0_, η_1_, η_2_, …, η_*K*_}. *q*_*j*0_ is fixed to 1 for each item, and η_0_ is the intercept. *e*_*j*_ is the residual of item *j* that cannot be explained by the *K* design variables, and $\sigma_{\varepsilon }^{2}$ is the residual variance. The explanatory rate of the item design variables (*R*^2^) for item difficulty in RELLTM is calculated as *R*^2^ = ($\sigma_{\beta }^{2}-\sigma_{\varepsilon }^{2}$)/$\sigma_{\beta }^{2}$, where $\sigma_{\beta }^{2}$ is the variance of difficulty across all items. When the *K* item design variables fully explain the variance of item difficulty, that is, *e*_*j*_= 0 for all items, RELLTM becomes LLTM. It should be noted that LLTM cannot calibrate the item difficulty directly; it needs to be predicted using the item design variables. When all regression coefficients (η_*k*_) and intercepts (η_0_) are fixed to 0, the RELLTM is equivalent to the Rasch model.

Hierarchical item family models include the Related Siblings Model (RSM; Sinharay et al., 2003), Unrelated Siblings Model (USM), and Identical Siblings Model (ISM). To be consistent with the item parameters of the cognitive IRT model, these three models are simplified versions that include only the item difficulty parameter. The formula for RSM is as follows:


$$\begin{array}{l} P\left(X_{ijl}\;=\;1|\theta_{i},\;b_{jl}\right)\;=\;\frac{\text{exp}\left(\theta_{i}\;-\;b_{jl}\right)}{1\;+\text{ exp}\left(\theta_{i}\;-\;b_{jl}\right)},\;b_{jl}\;\sim \;N\left(\xi_{l},\sigma_{l}^{2}\right). \end{array}$$

It is assumed that there are *L* different item families in the item bank, and each item belongs to only one item family. The *j*_*l*_ denotes the test item *j* that belongs to the item family *l* (*l* =1, 2,..., *L*). Since different items in the same item family share the same item generation rules, it can be assumed that there is a connection between all items belonging to item family *l*. This connection can be due to the fact that the item difficulty parameter *b* follows a normal distribution *N*(ξ_*l*_, $\sigma_{l}^{2}$), where ξ_*l*_ and $\sigma_{l}^{2}$ are the mean and the variance of this distribution, respectively. When there is no second level, $b_{jl}\;\sim \;N\left(\xi_{l},\sigma_{l}^{2}\right)$, the model is USM, which is equivalent to the Rasch model. When set $\sigma_{l}^{2}=$ 0 for all item families, the model is ISM.

In summary, there are five models (USM, ISM, RSM, RELLTM, and LLTM) for item difficulty calibration or prediction, with the first four belonging to calibrating item difficulty and LLTM to predicting item difficulty. In addition, USM/Rasch was used as a baseline model for comparison.

### 2.3 Simulation process

Two research questions mentioned above were explored through Monte Carlo simulation, and the simulation process is divided into four steps.

Step 1: Simulating an item bank in CAT based on the cognitive design system approach

First, this study assumes that all items in the item bank of CAT are consistent with the RELLTM model. According to the parameter settings of the RELLTM, the number of item families in the item bank (e.g., 30 item families) and the number of items within each item family need to be given, where the number of items within each item family is fixed to be equal for simplicity (e.g., each item family contains 10 items). Next, the number of item design variables (e.g., three design variables) and the level of each design variable need to be given. There is a restriction that the levels of these design variables are multiplied to equal the total number of item families. When the total number of item families is 30, this study sets the number of levels of design variables to 2, 3, and 5, respectively.

Then, it is also necessary to construct the *Q*-matrix of the item bank, which represents the association of items with item design variables. The specific *Q*-matrix (300 × 3) is shown in the Supplementary material, where every 10 rows represent an item family, and each item family represents a combination of item design variables. In addition, the regression coefficients (η_*k*_) and intercept (η_0_) need to be determined. To ensure the item design difficulty ($\Sigma_{k=0}^{K}q_{jk}\eta_{k}$) is in the range between −3 and 3, the intercept η_0_is set to −3. For simplicity, if we fix the multiplication of each design variable and the regression coefficients to be equal to 2, the regression coefficients η_1_, η_2_, and η_3_are set to 2, 1, and 0.5, respectively.

**Figure 1:**
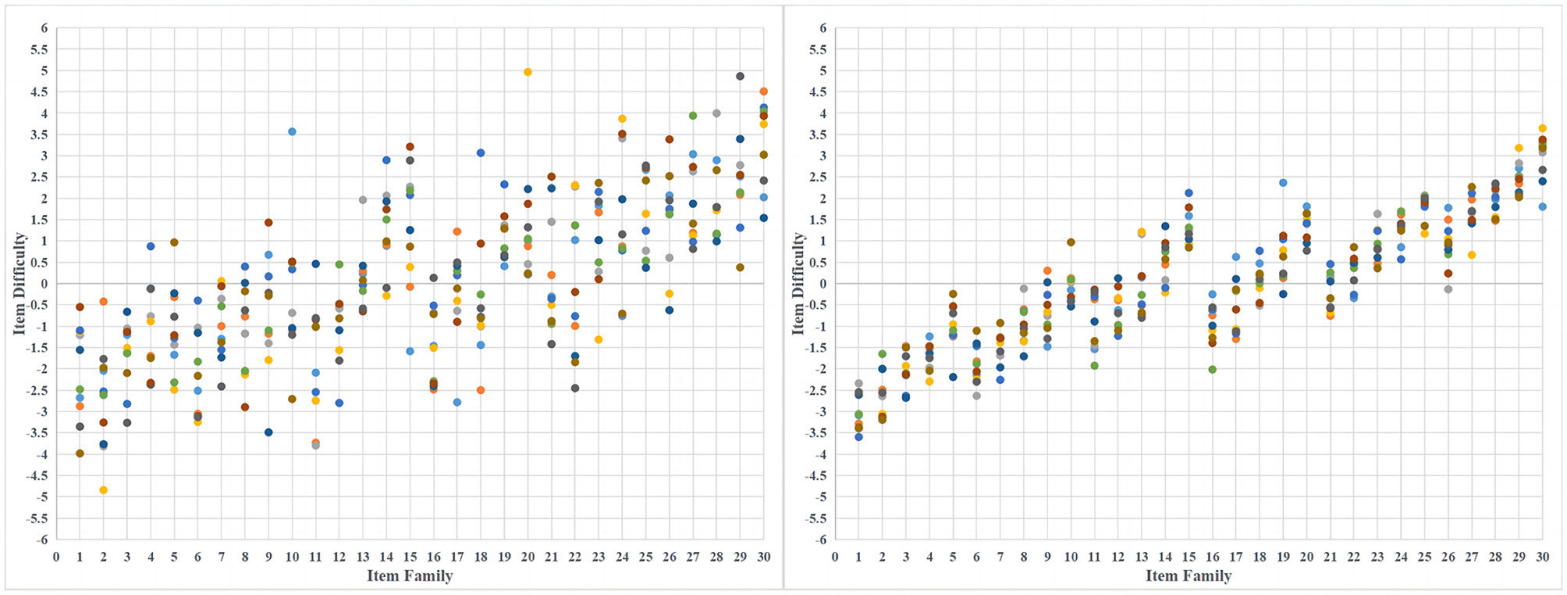
The simulated item difficulty distributions for *R*^2^ are 60% **(left)** and 90% **(right)**.

Finally, the true value of item difficulty *b* is the item design difficulty for each item plus the residual term *e*_*j*_. The residual term *e*_*j*_~*N*(0, $\sigma_{\varepsilon }^{2}$) and the determination of $\sigma_{\varepsilon }^{2}$ need to be calculated according to *R*^2^. The formula is *R*^2^ = ($\sigma_{\beta }^{2}$- $\sigma_{\varepsilon }^{2}$)/$\sigma_{\beta }^{2}=\sigma_{\tau }^{2}$/($\sigma_{\tau }^{2}+$ $\sigma_{\varepsilon }^{2}$), where $\sigma_{\tau }^{2}$ is the between-group variance (the variance between item design difficulty). In the case above, the value of $\sigma_{\tau }^{2}$ is 2.241. When the *R*^2^ is 60, 70, 80, and 90%, the value of $\sigma_{\varepsilon }^{2}$ can be calculated by the above formula to be 1.494, 0.961, 0.560, and 0.249, respectively. Therefore, when the *R*^2^ is set to 60%, the residual term *e*_*j*_ for each item is drawn from the normal distribution *N*(0, 1.494), where 1.494 is the variance. Figure 1 shows the simulated item difficulty distributions for *R*^2^ of 60 and 90%, respectively. Each point represents an item, and the colors are only used to distinguish between different items within the same item family. The difficulty for each item in Figure 1 is shown in the Supplementary material.

Step 2: Simulation of examinees' abilities and scores

The number of examinees was set to 1,000, and each examinee's ability (θ) was drawn from a standard normal distribution *N*(0, 1^2^).

The score matrix was generated using the RELLTM model. The correct response probability matrix for all item examinees is obtained by bringing the θ into the RELLTM model along with the item design parameters (η_*k*_, *q*_*jk*_, and *e*_*j*_) from Step 1. Each value of this correct response probability matrix is then compared with a random number drawn from the uniform distribution *Uniform*(0, 1). When the value is greater than or equal to the random number, the element in the corresponding position of the score matrix is 1. Otherwise, it is 0. In this way, the score matrix is generated.

Step 3: Estimation of item difficulty in the item bank

Although the true value of item difficulty was known, this study aimed to compare the effects of different ways of calibrating (or predicting) item difficulty on the measurement efficiency in CAT. In other words, the item difficulty needs to be re-parameterized in the same situation. Therefore, the same score matrix obtained in Step 2 was given to five different item difficulty calibration (or prediction) approaches. These five approaches are USM, ISM, RSM, RELLTM, and LLTM, as mentioned in Section 2.2.

The hierarchical item family model and the cognitive IRT model are not the same. To unify the process of estimation algorithmically, this study used a Markov Chain Monte Carlo (MCMC) parameter estimation procedure via R and RStan. To ensure that each model converged sufficiently, the criterion for Rhat in this study was set to be < 1.05. In the case of 1,000 examinees and 300 items, two MCMC chains were set up, and each chain was run 10,000 times, with the mean of the last 5,000 taken as the parameter estimates. Since then, five-item difficulty parameters have been estimated and used for subsequent item selection.

Step 4: Simulation of the CAT process

The CAT simulation process was accomplished through the R program. First, the initial item was selected by randomly selecting a moderately difficult item (difficulty between −0.1 and 0.1) from the item bank. Then, the examinee's score on the initial item was found in the score matrix in Step 2. Afterward, the examinee ability estimate (θ) on the Rasch model was estimated using the expected a posteriori (EAP) estimation method.

Then, the loop of item selection and scoring was entered according to the five-item difficulty calibrations (or predictions) methods. For example, when using item difficulty calibrated through the USM model, a specific item selection strategy (e.g., Maximum Fisher Information) was used to select the item that best fits the examinee's current ability estimate. It should be noted that the item difficulty used in item selection is the calibrated (or predicted) item difficulty. After selecting an item, the examinee's score on that item was found in the score matrix in Step 2. The EAP method was then used to estimate on the Rasch model. In this way, the item selection and scoring loop were carried out.

Finally, the stopping rule of the loop was a fixed test length; that is, the test ended after a fixed number of test items (e.g., 60 items). The final estimate of θ on the Rasch model using the EAP method was used as the CAT estimate of this examinee's ability.

### 2.4 Simulation design

To compare the performance of the five calibration (or prediction) procedures (USM, ISM, RSM, RELLTM, and LLTM) under different item banks, the variables indicated above were kept constant, and the variable *R*^2^ was manipulated (60, 70, 80, and 90%).

The dependent variables are the recovery of theta and the measurement efficiency of CAT. RMSE was used to represent the recovery of theta in CAT. Measurement efficiency in this CAT was evaluated by determining the number of additional test items required to achieve the same RMSE achieved by the baseline model (USM/Rasch). To thoroughly examine these metrics, a total of 20 experimental conditions were formed and repeated 50 times for each experimental condition.

## 3 Results

**Figure 2:**
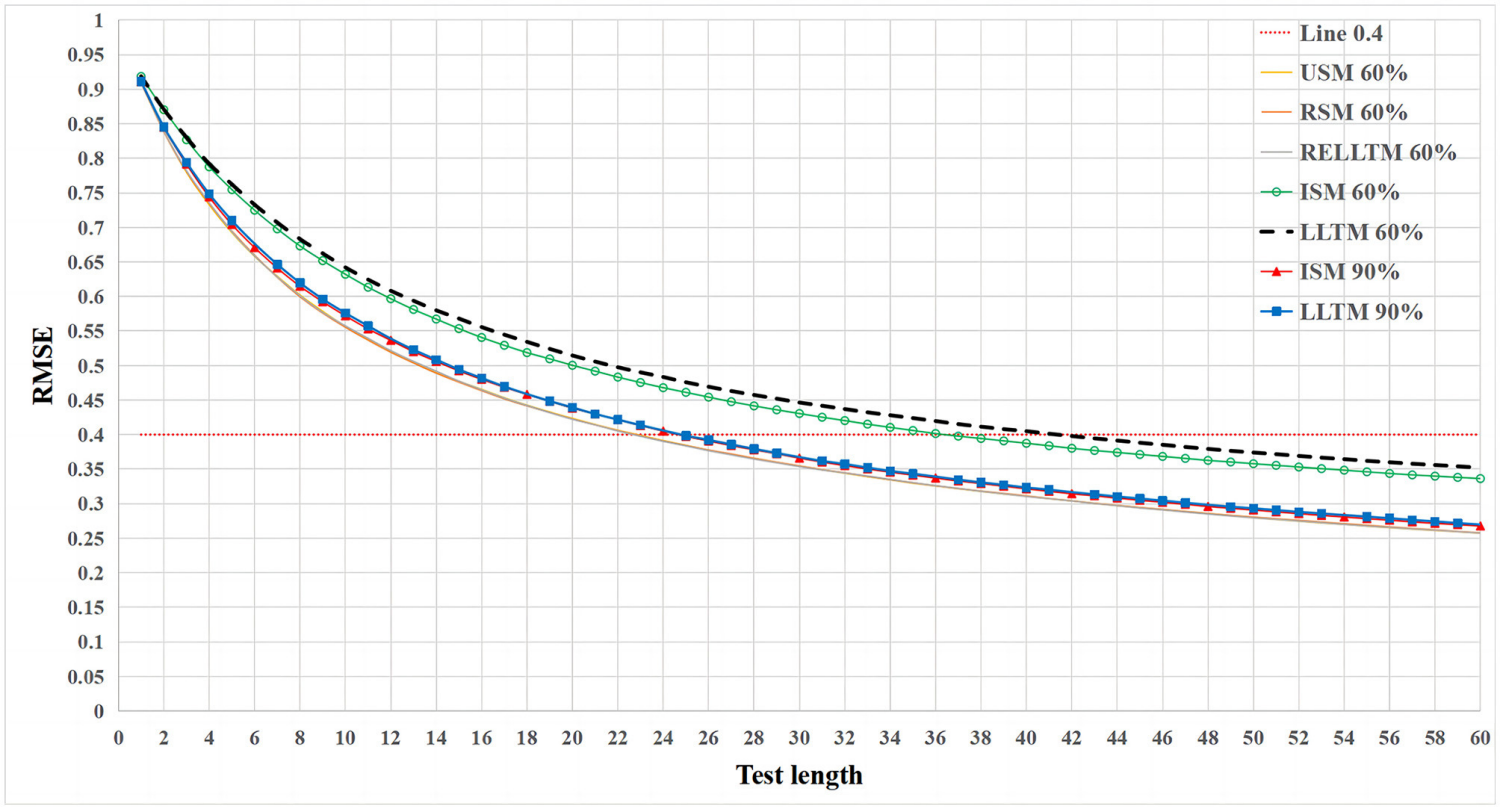
The variation of RMSE with test length for multiple experimental conditions at different *R*^2^.

For the first research question, the variation of RMSE with test length is shown in Figure 2.

Figure 2 only shows 7 of the 20 experimental conditions (USM 60%, RSM 60%, RELLTM 60%, ISM 60%, LLTM 60%, ISM 90%, and LLTM 90%). The appropriate simplification is made because the curves of USM, RSM, and RELLTM overlap for all four cases with an *R*^2^ of 60, 70, 80, and 90%. The remaining conditions (ISM 70%, LLTM 70%, ISM 80%, and LLTM 80%) were not painted for simplicity. Figure 2 shows that (1) the RMSE for USM, RSM, and RELLTM always remained equal, regardless of the level of the *R*^2^ and the test length and that (2) USM, RSM, and RELLTM are similar classes of curves, while ISM and LLTM are other classes. The RMSE of ISM and LLTM is always larger than other classes (USM, RSM, and RELLTM) regardless of test length, and the gap decreases as the *R*^2^ increases. When the *R*^2^ reaches 90%, the gap between the curves of the two classes is very small. (3) There is also a gap between ISM and LLTM, with LLTM having a higher RMSE than ISM, but the difference is not notable. In particular, the two curves overlap when the *R*^2^ increases to 90%.

More specifically, Table 1 demonstrates the RMSE when the test length is 30. A portion of the table shows the RMSE for the 20 experimental conditions, and the last two columns show the difference between the LLTM and the other two models in RMSE. Since each experimental design was repeated 50 times, differences between these models could be evaluated using ANOVA or independent sample *t*-tests. First, Table 1 also shows no difference between USM, RSM, and RELLTM in RMSE. Second, the independent sample *t*-test shows that the RMSE of LLTM is significantly higher than that of ISM when *R*^2^ = 60, 70, and 80%, but the difference with ISM is not significant when *R*^2^ = 90% (*t* = 0.935, *p* = 0.353, *Cohen's d* = 0.187). Finally, the independent sample *t*-test shows that the RMSE of LLTM is significantly higher than that of USM when *R*^2^ = 60, 70, 80, and 90%.

**Table 1:** RMSE and RMSE difference for five calibration (or prediction) methods at different *R*^2^.

***R* ^2^**	**USM**	**RSM**	**RELLTM**	**ISM**	**LLTM**	**LLTM-ISM**	**LLTM-USM**
60%	0.354	0.355	0.354	0.431	0.446	0.015^***^ (1.118)	0.092^***^ (7.020)
70%	0.355	0.356	0.357	0.405	0.418	0.013^***^ (1.124)	0.063^***^ (5.866)
80%	0.353	0.355	0.355	0.382	0.392	0.010^***^ (1.006)	0.039^***^ (4.339)
90%	0.354	0.356	0.355	0.366	0.367	0.001 (0.187)	0.013^***^ (1.625)

^***^Represents *p* < 0.001. The numbers in parentheses in the last two columns are *Cohen's d*.

**Figure 3:**
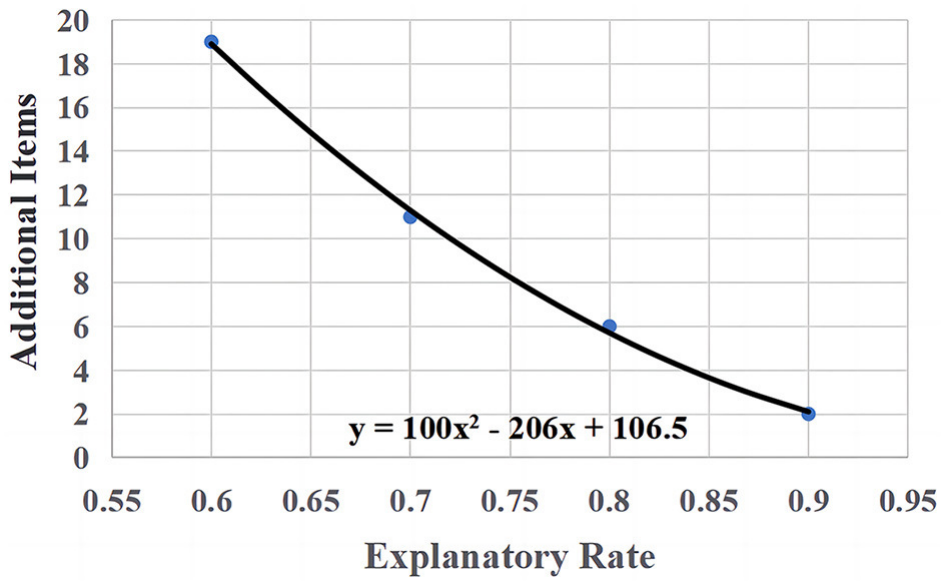
The relationship between the number of additional items and the *R*^2^ when RMSE = 0.4 is used as the criterion.

For the second research question, the model of interest is the LLTM. As can be seen in Figure 2, the gap between RMSE under LLTM (predicted difficulty) and baseline model USM (calibrated difficulty) decreases as *R*^2^ increases. The LLTM is worse than USM in terms of RMSE, and the LLTM requires several additional items to achieve the same RMSE as USM. With the same RMSE criterion (RMSE = 0.4), the LLTM was compared to the baseline model (USM/Rasch), and Figure 3 shows the relationship between the number of additional items in CAT needed for the LLTM and the *R*^2^. Figure 3 describes a monotonically decreasing quadratic curve with the curve equation y = 100x^2^ – 206x + 106.5. If one wishes the test length of the CAT based on the predicted difficulty to be no more than 30 (including eight additional items), at least 75% of the *R*^2^ is needed.

## 4 Discussion

This study compared the effects of five-item calibration (or prediction) approaches on measurement efficiency in CAT. In response to the first research question, we obtained the following four conclusions: (1) These five approaches were divided into two categories in terms of measurement efficiency, and the first category (USM, RSM, and RELLTM) outperforms the second category (LLTM and ISM); (2) the RMSE of the first category does not vary with the *R*^2^. The RMSE of the second category decreases with *R*^2^, and the gap with the RMSE of the first category decreases accordingly; (3) in the second category (LLTM and ISM), ISM performs better on RMSE than LLTM; and (4) in response to the second research question, we conclude that the predictive model (LLTM) is worse than the baseline model (USM) in terms of RMSE, but the LLTM only needs a few more items to produce the same RMSE as the baseline model and that the number of additional items decreases as the *R*^2^ increases.

For Conclusion (1), USM, RSM, and RELLTM do not differ in RMSE because all three models have separate estimates of item difficulty, with RELLTM having separate estimates of the residual term *e*_*j*_ for each item difficulty. In contrast, LLTM and ISM were only estimated at the item family level, and item difficulty within the item family was not estimated individually, which is simpler for the former category. Therefore, the first category is better than the second category in terms of measurement efficiency in CAT. Conclusion (2) is also in line with our expectations. As the *R*^2^ increases, it means that the data are increasingly consistent with the LLTM and ISM models, while the other three models are not affected by the *R*^2^ because they have separate estimates of item difficulty.

For conclusion (3), it is established that the LLTM is a generalized linear fixed-effects model in which item difficulty is predicted from item design variables. Therefore, in estimating item difficulty, compared to the ISM model, the LLTM model exhibits the phenomenon of regressing to the mean of the difficulty parameter of test items (regression to the mean). The degree of regression primarily depends on the validity of the item cognitive model and the quality of the item design features (Embretson, 1999). However, each item's difficulty was estimated separately in the ISM, so there is no phenomenon of “regression to the mean.” It is worth noting that the gap caused by this phenomenon of “regression to the mean” decreases as the *R*^2^ increases. A high *R*^2^ indicates that the difficulty of items within the item family tends to be more consistent. This finding means that the estimated regression coefficient for the item design variables in the LLTM is more stable and effective, leading to a more accurate prediction of item difficulty.

Conclusion (4) has significant value for item generation in CAT, especially for CAT, where items are generated on the fly. Item generation on the fly means that the items are generated instantaneously. Item difficulty can only be used with predictive difficulty. The results of the LLTM predictive difficulty tell us how many additional test items are needed in CAT to achieve the same RMSE as the baseline model or at least how much *R*^2^ is guaranteed to compensate for the loss of measurement efficiency from using predictive difficulty.

Finally, this study constructed a CAT item bank based on the CDS by means of simulation. The simulated item bank is not a substitute for a real-item bank, but the simulation approach allows for a wide variety of item bank situations to be easily obtained. We also used a real item bank for validation. All the items in this real item bank were mental rotation items measuring spatial ability, and the item bank was constructed using the CDS approach. The results under the real item bank are consistent with those under the simulated item bank.

With the advent of artificial intelligence (AI), item generation based on AI will become more and more common. However, this is only a technological advancement; validity is still key to item generation. Validity involves theoretical thinking about the construct, which is difficult to achieve with the current form of AI. Recent studies have used GPT for item generation for personality items (Hommel et al., 2022), which is more like a weak-theory item generation approach, and item generation methods that combine GPT with a cognitive design system approach still need to be developed. In conclusion, CAT based on the CDS approach is highly promising and practical. It combines cognitive psychology, psychometrics, and computer science and is one of the future directions of the new generation of AI assessment.

## Data availability statement

The raw data supporting the conclusions of this article will be made available by the authors, without undue reservation.

## Author contributions

HL: Writing – original draft, Writing – review & editing. XY: Writing – review & editing.
