# Supplementary material for: Efficiency of computerized adaptive testing with a cognitively designed item bank
Source: Front Psychol. 2024 Jun 26;15:1353419. doi: 10.3389/fpsyg.2024.1353419 (PMC11233769; doi:10.3389/fpsyg.2024.1353419)
Supplement: Supplementary file 1 [file Data_Sheet_1.docx]

**Supplementary Material**

Table A1. Q-matrix between item (item family) and item design variables.

| Item | Item family | Item design variables | | |
| --- | --- | --- | --- | --- |
|  |  | *K1* | *K2* | *K3* |
| 1-10 | 1 | 0 | 0 | 0 |
| 11-20 | 2 | 0 | 0 | 1 |
| 21-30 | 3 | 0 | 0 | 2 |
| 31-40 | 4 | 0 | 0 | 3 |
| 41-50 | 5 | 0 | 0 | 4 |
| 51-60 | 6 | 0 | 1 | 0 |
| 61-70 | 7 | 0 | 1 | 1 |
| 71-80 | 8 | 0 | 1 | 2 |
| 81-90 | 9 | 0 | 1 | 3 |
| 91-100 | 10 | 0 | 1 | 4 |
| 101-110 | 11 | 0 | 2 | 0 |
| 111-120 | 12 | 0 | 2 | 1 |
| 121-130 | 13 | 0 | 2 | 2 |
| 131-140 | 14 | 0 | 2 | 3 |
| 141-150 | 15 | 0 | 2 | 4 |
| 151-160 | 16 | 1 | 0 | 0 |
| 161-170 | 17 | 1 | 0 | 1 |
| 171-180 | 18 | 1 | 0 | 2 |
| 181-190 | 19 | 1 | 0 | 3 |
| 191-200 | 20 | 1 | 0 | 4 |
| 201-210 | 21 | 1 | 1 | 0 |
| 211-220 | 22 | 1 | 1 | 1 |
| 221-230 | 23 | 1 | 1 | 2 |
| 231-240 | 24 | 1 | 1 | 3 |
| 241-250 | 25 | 1 | 1 | 4 |
| 251-260 | 26 | 1 | 2 | 0 |
| 261-270 | 27 | 1 | 2 | 1 |
| 271-280 | 28 | 1 | 2 | 2 |
| 281-290 | 29 | 1 | 2 | 3 |
| 291-300 | 30 | 1 | 2 | 4 |

**Brief description of developing a mental rotation CAT item bank using the Cognitive Design**

**System approach**

There are several steps in developing a CAT item bank using the Cognitive Design System approach.

Step 1: Identify Measured Construct and Item Type.

The measured construct by the mental rotation item is spatial ability and the item type uses the mental rotation item developed by Shepard and Metzler (1971), shown in Figure A1. Subjects were required to mentally imagine rotating one of the objects to determine whether the left and right figures were the same.

Step 2: Cognitive Model of the Items.

The cognitive model for solving the mental rotation item is the cognitive process model proposed by Lohman and Ippel (1993), as shown in Figure A2.

Step 3: Identify construct-relevant design variables and construct-irrelevant design variable.

Based on the relevant literature (Shepard & Metzler, 1971; Peters et al., 1995), this paper identifies four construct-relevant design variables (figure complexity, rotation type, rotation angle, and whether mirroring) and one construct-irrelevant design variable (drawing mode). See Figure A1 for details.

Step 4: Formation of Item Generation Algorithms.

First, four construct-relevant design variables can be combined to form several item generation rules (78 rules). Then, algorithmic or automated generation of a large number of items is achieved by changing construct-irrelevant design variables under the same item generation rule.

Step 5: Item Generation and Measurement.

A total of 624 items were generated by combining these design variables. These items were drawn using ShetchUp and Photoshop software. 624 items were administered in the form of subtests to 3864 primary and secondary school students.

Step 6: Testing of Item Design Variables.

The validity of the item design variables was tested through the Random Effect LLTM model. As shown in Tables A2 and A3, the results showed that all four construct-relevant design variables significantly affected item difficulty, and these four design variables explained 72.6% of the variation in item difficulty. Finally, the difficulty scatterplot of the 624 items under the 78 item generation rule is shown in Figure A3.

Step 7: Testing of Item Measurement Characteristics

In this article, the testing of item measurement characteristics is divided into testing of item fit and testing of item parameter distributions. The item fit statistics *S*-*X*^2^ (Orlando& Thissen, 2000) of each item is calculated in flexMIRT, and only 43 items (6.9% items) fitted the Rasch model worse (the *p* value corresponding to the *S*-*X*^2^ is less than 0.001). It indicates that the actual response data is consistent with Rasch model. On the other hand, the histogram of the item bank difficulty frequency distribution on the Rasch model is shown in Figure A4. Overall, the item difficulty frequency distribution is closer to the normal curve. Specifically, the range of item difficulty is -2.46 to 2.03 (*Mean*= -0.04, *SD*= 0.91, *Skewness*= -0.26, *Kurtosis*= -0.52), with a wide range of difficulty coverage.

**Simulation experiment on measurement efficiency of different item calibration methods under mental rotation CAT item bank**

**Methods**

Unlike the simulation experiment in the “Developing Item Bank with Cognitive Design System Approach” section, the item bank for the simulation experiment here is the real mental rotation CAT item bank, which was also constructed based on the Cognitive Design System approach. From the previous subsection, it can be seen that both the response data on the real item bank and the response data on the simulated item bank conform to the Rasch model, and the difficulty distributions under both the real item bank and the simulated item bank approximately obey a normal distribution. This all indicates that the simulated item bank is consistent with the real item bank.

In the remaining section “Models for calibrating or predicting item difficulty”, the real item bank is consistent with the simulated item bank. In the “Simulation Process” section, the simulation process based on the real item bank is fixed in terms of the difficulty of the item bank and the explanatory rate, but otherwise remains constant. In the “Simulation Design” section, the simulation design based on the real item bank was fixed in terms of the explanatory rate (*R*^2^=72.6%), so only five experimental conditions were formed.

**Results**

Figure A5 below shows the trend of RMSE with test length for four item difficulty calibration methods (USM, ISM, RSM, and RELLTM) and one item difficulty prediction method (LLTM) under the real item bank. From Figure A5, it can be seen that: (1) Under the real CAT item bank with 72.6% explanatory rate, the USM, RSM, and RELLTM are always consistent regardless of the test length. That is, the three curves always overlap in this figure, so no markers were added to the curves to distinguish between them. (2) RMSE under LLTM was the worst, followed by ISM. An independent samples *t*-test showed that RMSE under LLTM was significantly higher than USM at the test length of 30 (*t*=12.364, *p*<0.001, *Cohen's d*=2.418). (3) Comparing the LLTM to the baseline calibration approach (USM/Rasch), under the same RMSE criterion (RMSE=0.4), the LLTM requires 4 (27-23=4) more items to achieve the same RMSE. If under the simulated item bank, the LLTM requires approximately 9 more items (see Figure 3) to achieve the same RMSE when the explanatory rate is 72.6%. The smaller number of additional items required under the real item bank compared to the simulated bank may be related to the increase in the total number of items in the real item bank. All of this shows that the same results were produced under the real item bank as under the simulated item bank.


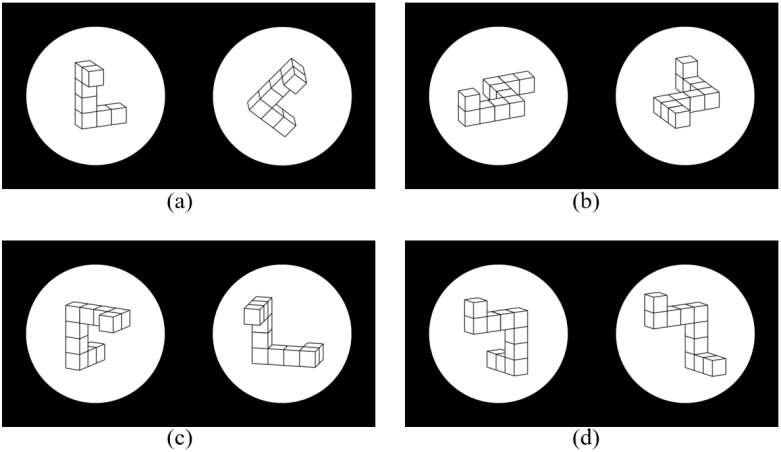


Figure A1. Four example diagrams of mental rotation items.

*Note*: Correspondence between design variables and graphs:

3 arms with 2 planes (a); 4 arms with 2 planes (b, d);

4 arms with 3 planes (c); solid-body rotation (b);

picture-plane rotation (a); mixed rotation (c);

mirroring process(b, d); no mirroring process (a, c).


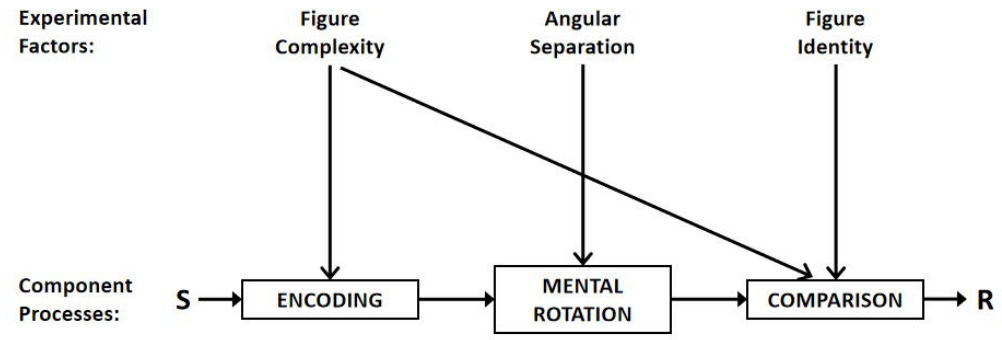


Figure A2. cognitive process model of the mental rotation item.

Table A2. Coefficients of design variables under the random-effect LLTM model.

| Design Variables and Intercept | *Coefficients η* | *Standard Error* | *z value* | *Sig.* |
| --- | --- | --- | --- | --- |
| Intercept | -3.548 | 0.091 | -39.026 | *p*<0.001 |
| whether mirroring | 1.102 | 0.040 | 27.673 | *p*<0.001 |
| rotation type |  |  |  |  |
| (no rotation) | 0 | — | — | — |
| (solid-body rotation) | 0.262 | 0.098 | 2.663 | *P=*0.008 |
| (picture-plane rotation) | 0.540 | 0.098 | 5.483 | *p*<0.001 |
| (mixed rotation) | 1.143 | 0.098 | 11.651 | *p*<0.001 |
| rotation angle | 0.178 | 0.018 | 9.624 | *p*<0.001 |
| figure complexity |  |  |  |  |
| (3 arms with 2 planes) | 0 | — | — | — |
| (4 arms with 2 planes) | 0.325 | 0.049 | 6.608 | *p*<0.001 |
| (4 arms with 3 planes) | 0.674 | 0.056 | 11.993 | *p*<0.001 |

Table A3. Degree of explanation of item design variables.

| Design Variables | *σ*2 θ | *σ*2 β | *σ*2 ε | *R*^2^ |
| --- | --- | --- | --- | --- |
| figure complexity, rotation type, rotation angle, and whether mirroring | 0.950 | 0.747 | 0.205 | 0.726 |


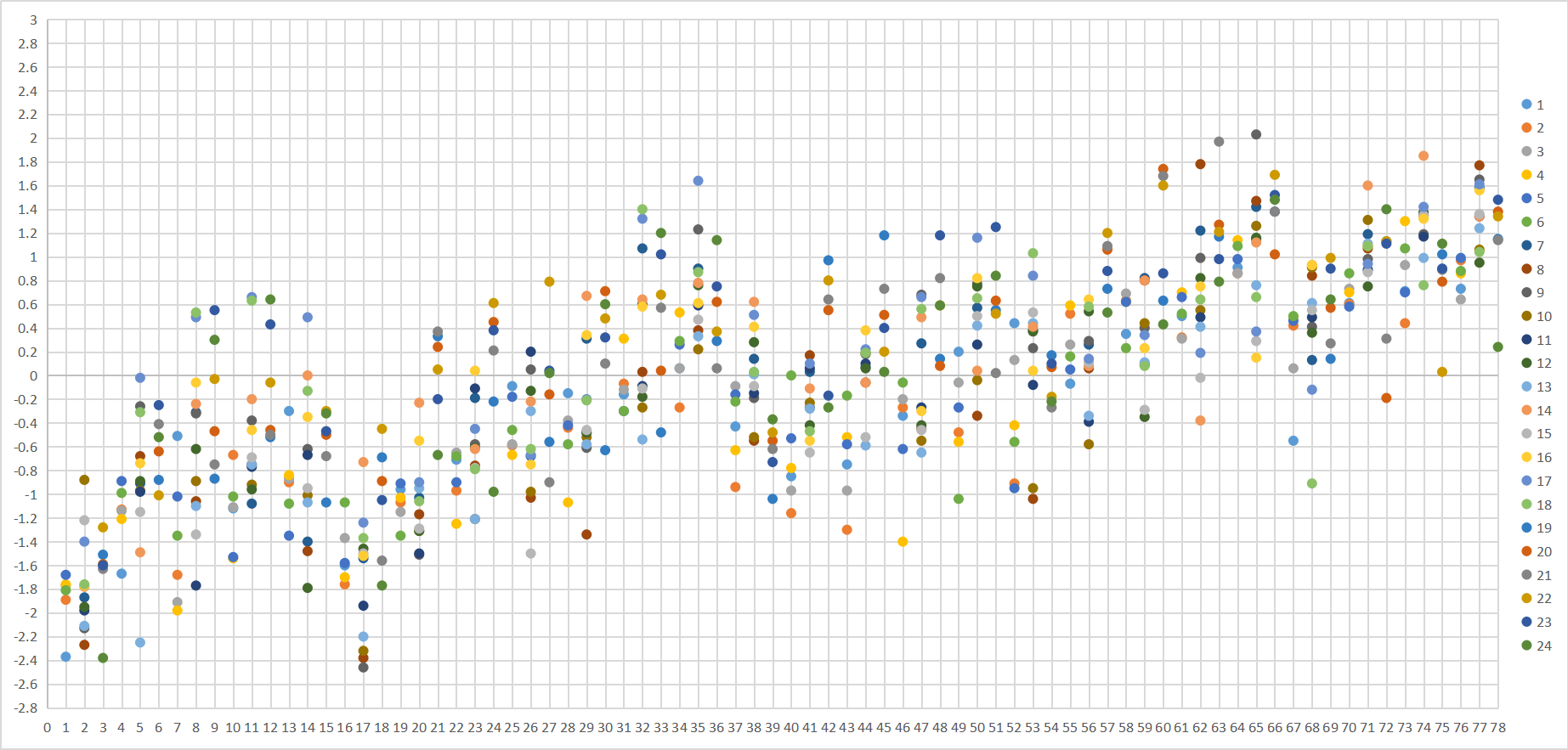


Figure A3. Scatterplot of mental rotation items.


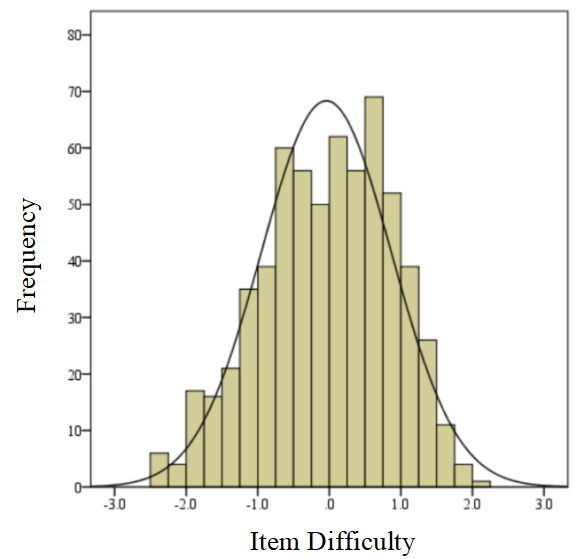


Figure A4. Histogram of the item bank difficulty frequency distribution.


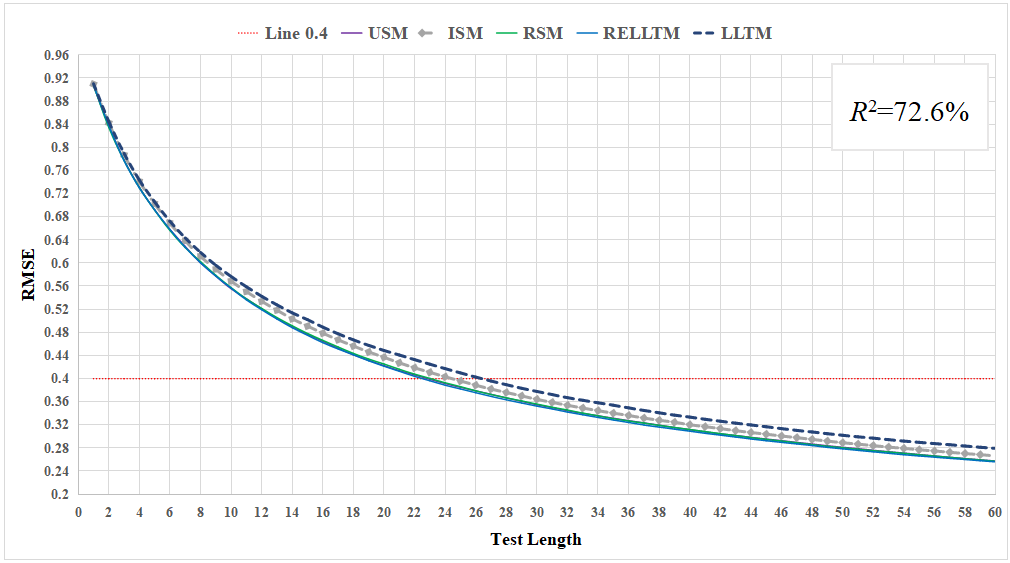


Figure A5. RMSE of CAT in different item difficulty calibration methods under mental rotation CAT item bank.

**References**

Lohman, D. F., & Ippel, M. J. (1993). Cognitive diagnosis: from statistically based assessment toward theory based assessment. In N. Frederiksen, R. J. Mislevy & I. I. Bejar (Eds.), *Test theory for a new generation of tests* (pp. 41−71). Hillsdale, NJ: Erlbaum.

Orlando, M., & Thissen, D. (2000). Likelihood-based item-fit indices for dichotomous item response theory models. *Applied Psychological Measurement*, *24*(1), 50-64.

Peters, M., Laeng, B., Latham, K., Jackson, M., Zaiyouna, R., & Richardson, C. (1995). A redrawn vandenberg and kuse mental rotations test: different versions and factors that affect performance. *Brain and Cognition*, *28*(1), 39−58.

Shepard, R. N., & Metzler, J. (1971). Mental rotation of three-dimensional objects. *Science*, *171*(3972), 701−703.

Table A4. The simulated item difficulty for *R*^2^ of 60% and 90%.

| Family | Item | Difficulty at  *R*^2^= 60% | Difficulty at  *R*^2^= 90% |  | Family | Item | Difficulty at  *R*^2^= 60% | Difficulty at  *R*^2^= 90% |
| --- | --- | --- | --- | --- | --- | --- | --- | --- |
| 1 | 1 | -2.679 | -3.088 |  | 16 | 151 | -1.459 | -0.250 |
| 1 | 2 | -2.875 | -3.283 |  | 16 | 152 | -2.484 | -0.742 |
| 1 | 3 | -1.206 | -2.340 |  | 16 | 153 | -0.683 | -0.638 |
| 1 | 4 | -1.132 | -2.606 |  | 16 | 154 | -1.510 | -1.097 |
| 1 | 5 | -1.096 | -3.598 |  | 16 | 155 | -0.516 | -0.607 |
| 1 | 6 | -2.482 | -3.059 |  | 16 | 156 | -2.289 | -2.015 |
| 1 | 7 | -1.556 | -2.607 |  | 16 | 157 | -2.404 | -0.987 |
| 1 | 8 | -0.548 | -3.377 |  | 16 | 158 | -2.344 | -1.393 |
| 1 | 9 | -3.353 | -2.543 |  | 16 | 159 | 0.135 | -0.556 |
| 1 | 10 | -3.982 | -3.393 |  | 16 | 160 | -0.713 | -1.269 |
| 2 | 11 | -2.041 | -2.005 |  | 17 | 161 | -2.783 | 0.626 |
| 2 | 12 | -0.420 | -2.489 |  | 17 | 162 | 1.221 | -1.300 |
| 2 | 13 | -3.817 | -2.641 |  | 17 | 163 | -0.638 | -1.072 |
| 2 | 14 | -4.843 | -3.056 |  | 17 | 164 | -0.404 | -1.067 |
| 2 | 15 | -2.530 | -3.146 |  | 17 | 165 | 0.195 | -1.168 |
| 2 | 16 | -2.613 | -1.649 |  | 17 | 166 | 0.303 | -0.171 |
| 2 | 17 | -3.766 | -1.998 |  | 17 | 167 | 0.418 | 0.108 |
| 2 | 18 | -3.257 | -3.124 |  | 17 | 168 | -0.897 | -0.607 |
| 2 | 19 | -1.769 | -2.557 |  | 17 | 169 | 0.497 | -1.118 |
| 2 | 20 | -1.972 | -3.196 |  | 17 | 170 | -0.113 | -0.135 |
| 3 | 21 | -1.201 | -2.150 |  | 18 | 171 | -1.441 | 0.473 |
| 3 | 22 | -1.621 | -1.465 |  | 18 | 172 | -2.499 | 0.026 |
| 3 | 23 | -1.050 | -1.490 |  | 18 | 173 | -1.006 | -0.517 |
| 3 | 24 | -1.509 | -1.929 |  | 18 | 174 | -0.978 | -0.103 |
| 3 | 25 | -2.820 | -2.632 |  | 18 | 175 | 3.068 | 0.769 |
| 3 | 26 | -1.633 | -2.102 |  | 18 | 176 | -0.254 | 0.008 |
| 3 | 27 | -0.661 | -2.679 |  | 18 | 177 | -0.781 | 0.207 |
| 3 | 28 | -1.132 | -2.133 |  | 18 | 178 | 0.940 | -0.453 |
| 3 | 29 | -3.264 | -1.704 |  | 18 | 179 | -0.579 | 0.102 |
| 3 | 30 | -2.098 | -1.500 |  | 18 | 180 | -0.810 | 0.234 |
| 4 | 31 | -0.112 | -1.240 |  | 19 | 181 | 0.408 | 2.366 |
| 4 | 32 | -1.699 | -1.522 |  | 19 | 182 | 0.676 | 0.128 |
| 4 | 33 | -0.766 | -1.969 |  | 19 | 183 | 1.373 | 0.233 |
| 4 | 34 | -0.888 | -2.296 |  | 19 | 184 | 1.285 | 0.782 |
| 4 | 35 | 0.876 | -1.472 |  | 19 | 185 | 2.328 | 1.045 |
| 4 | 36 | -2.373 | -1.746 |  | 19 | 186 | 0.828 | 0.176 |
| 4 | 37 | -2.362 | -1.637 |  | 19 | 187 | 0.620 | -0.245 |
| 4 | 38 | -2.327 | -1.470 |  | 19 | 188 | 1.578 | 1.124 |
| 4 | 39 | -0.123 | -1.740 |  | 19 | 189 | 0.667 | 0.235 |
| 4 | 40 | -1.744 | -2.044 |  | 19 | 190 | 1.294 | 0.634 |
| 5 | 41 | -1.670 | -1.239 |  | 20 | 191 | 1.013 | 1.812 |
| 5 | 42 | -0.315 | -1.222 |  | 20 | 192 | 0.880 | 1.629 |
| 5 | 43 | -1.434 | -0.574 |  | 20 | 193 | 0.458 | 1.399 |
| 5 | 44 | -2.489 | -0.956 |  | 20 | 194 | 4.963 | 1.518 |
| 5 | 45 | -1.276 | -1.198 |  | 20 | 195 | 0.219 | 1.420 |
| 5 | 46 | -2.318 | -1.094 |  | 20 | 196 | 1.052 | 1.647 |
| 5 | 47 | -0.225 | -2.191 |  | 20 | 197 | 2.219 | 0.951 |
| 5 | 48 | -1.211 | -0.530 |  | 20 | 198 | 1.872 | 1.082 |
| 5 | 49 | -0.775 | -0.697 |  | 20 | 199 | 1.321 | 0.774 |
| 5 | 50 | 0.967 | -0.242 |  | 20 | 200 | 0.241 | 1.646 |
| 6 | 51 | -2.512 | -2.135 |  | 21 | 201 | -0.304 | 0.166 |
| 6 | 52 | -3.052 | -1.825 |  | 21 | 202 | 0.200 | -0.756 |
| 6 | 53 | -1.033 | -2.632 |  | 21 | 203 | 1.451 | 0.122 |
| 6 | 54 | -3.247 | -2.247 |  | 21 | 204 | -0.502 | -0.677 |
| 6 | 55 | -0.398 | -1.463 |  | 21 | 205 | -0.355 | 0.458 |
| 6 | 56 | -1.830 | -1.884 |  | 21 | 206 | -0.945 | 0.259 |
| 6 | 57 | -1.153 | -1.404 |  | 21 | 207 | 2.236 | 0.054 |
| 6 | 58 | -3.106 | -2.060 |  | 21 | 208 | 2.510 | -0.576 |
| 6 | 59 | -3.130 | -2.301 |  | 21 | 209 | -1.419 | -0.551 |
| 6 | 60 | -2.163 | -1.107 |  | 21 | 210 | -0.879 | -0.344 |
| 7 | 61 | -1.295 | -1.300 |  | 22 | 211 | 1.023 | -0.339 |
| 7 | 62 | -0.998 | -1.260 |  | 22 | 212 | -0.992 | 0.445 |
| 7 | 63 | -0.355 | -1.687 |  | 22 | 213 | 2.273 | 0.523 |
| 7 | 64 | 0.058 | -1.388 |  | 22 | 214 | 2.309 | 0.527 |
| 7 | 65 | -1.551 | -2.256 |  | 22 | 215 | -0.763 | -0.262 |
| 7 | 66 | -0.530 | -1.289 |  | 22 | 216 | 1.366 | 0.366 |
| 7 | 67 | -1.730 | -1.963 |  | 22 | 217 | -1.699 | 0.488 |
| 7 | 68 | -0.060 | -1.273 |  | 22 | 218 | -0.197 | 0.584 |
| 7 | 69 | -2.412 | -1.590 |  | 22 | 219 | -2.452 | 0.077 |
| 7 | 70 | -1.376 | -0.924 |  | 22 | 220 | -1.842 | 0.856 |
| 8 | 71 | -2.067 | -1.339 |  | 23 | 221 | 1.831 | 1.255 |
| 8 | 72 | -0.772 | -0.602 |  | 23 | 222 | 1.672 | 0.477 |
| 8 | 73 | -1.172 | -0.119 |  | 23 | 223 | 0.281 | 1.636 |
| 8 | 74 | -2.133 | -1.357 |  | 23 | 224 | -1.312 | 1.257 |
| 8 | 75 | 0.399 | -0.650 |  | 23 | 225 | 2.153 | 1.238 |
| 8 | 76 | -2.047 | -0.666 |  | 23 | 226 | 0.498 | 0.935 |
| 8 | 77 | 0.017 | -1.706 |  | 23 | 227 | 1.020 | 0.611 |
| 8 | 78 | -2.897 | -0.960 |  | 23 | 228 | 0.103 | 0.803 |
| 8 | 79 | -0.626 | -1.055 |  | 23 | 229 | 1.925 | 0.820 |
| 8 | 80 | -0.179 | -1.156 |  | 23 | 230 | 2.361 | 0.361 |
| 9 | 81 | 0.675 | -1.478 |  | 24 | 231 | -0.758 | 0.855 |
| 9 | 82 | -1.176 | 0.304 |  | 24 | 232 | 0.875 | 1.621 |
| 9 | 83 | -1.400 | -0.757 |  | 24 | 233 | 3.406 | 1.260 |
| 9 | 84 | -1.795 | -0.663 |  | 24 | 234 | 3.868 | 1.322 |
| 9 | 85 | 0.169 | -0.263 |  | 24 | 235 | 0.781 | 0.570 |
| 9 | 86 | -1.098 | -0.960 |  | 24 | 236 | 0.826 | 1.695 |
| 9 | 87 | -3.488 | 0.033 |  | 24 | 237 | 1.980 | 1.406 |
| 9 | 88 | 1.432 | -0.497 |  | 24 | 238 | 3.513 | 1.392 |
| 9 | 89 | -0.215 | -1.291 |  | 24 | 239 | 1.156 | 1.344 |
| 9 | 90 | -0.284 | -1.041 |  | 24 | 240 | -0.705 | 1.244 |
| 10 | 91 | 3.566 | -0.148 |  | 25 | 241 | 2.670 | 2.042 |
| 10 | 92 | -1.092 | 0.127 |  | 25 | 242 | 0.530 | 1.841 |
| 10 | 93 | -0.688 | 0.024 |  | 25 | 243 | 0.771 | 2.070 |
| 10 | 94 | 0.508 | 0.077 |  | 25 | 244 | 1.639 | 1.171 |
| 10 | 95 | 0.338 | -0.399 |  | 25 | 245 | 1.239 | 1.806 |
| 10 | 96 | 0.487 | 0.095 |  | 25 | 246 | 0.537 | 2.047 |
| 10 | 97 | -1.042 | -0.535 |  | 25 | 247 | 0.373 | 1.967 |
| 10 | 98 | 0.517 | -0.312 |  | 25 | 248 | 2.722 | 1.906 |
| 10 | 99 | -1.200 | -0.416 |  | 25 | 249 | 2.769 | 2.009 |
| 10 | 100 | -2.707 | 0.971 |  | 25 | 250 | 2.420 | 1.353 |
| 11 | 101 | -2.089 | -1.536 |  | 26 | 251 | 2.069 | 1.778 |
| 11 | 102 | -3.733 | -0.366 |  | 26 | 252 | 1.724 | 1.500 |
| 11 | 103 | -3.799 | -1.455 |  | 26 | 253 | 0.607 | -0.132 |
| 11 | 104 | -2.745 | -0.278 |  | 26 | 254 | -0.238 | 1.053 |
| 11 | 105 | -2.544 | -0.304 |  | 26 | 255 | 1.755 | 1.234 |
| 11 | 106 | -1.000 | -1.925 |  | 26 | 256 | 1.625 | 0.688 |
| 11 | 107 | 0.464 | -0.889 |  | 26 | 257 | -0.623 | 0.797 |
| 11 | 108 | -0.843 | -0.139 |  | 26 | 258 | 3.385 | 0.240 |
| 11 | 109 | -0.807 | -0.204 |  | 26 | 259 | 1.956 | 0.906 |
| 11 | 110 | -1.016 | -1.349 |  | 26 | 260 | 2.522 | 0.969 |
| 12 | 111 | -0.552 | -0.623 |  | 27 | 261 | 3.036 | 1.667 |
| 12 | 112 | -0.517 | -0.389 |  | 27 | 262 | 1.193 | 1.973 |
| 12 | 113 | -0.584 | -1.010 |  | 27 | 263 | 2.632 | 1.681 |
| 12 | 114 | -1.560 | -0.339 |  | 27 | 264 | 1.131 | 0.671 |
| 12 | 115 | -2.799 | -1.224 |  | 27 | 265 | 0.983 | 2.119 |
| 12 | 116 | 0.451 | -0.971 |  | 27 | 266 | 3.940 | 1.453 |
| 12 | 117 | -1.094 | 0.125 |  | 27 | 267 | 1.875 | 1.415 |
| 12 | 118 | -0.475 | -0.066 |  | 27 | 268 | 2.739 | 1.492 |
| 12 | 119 | -1.805 | -0.695 |  | 27 | 269 | 0.816 | 1.703 |
| 12 | 120 | -0.814 | -1.100 |  | 27 | 270 | 1.408 | 2.267 |
| 13 | 121 | 0.231 | 0.145 |  | 28 | 271 | 2.895 | 1.973 |
| 13 | 122 | 0.295 | -0.511 |  | 28 | 272 | 1.155 | 1.475 |
| 13 | 123 | 1.965 | 1.164 |  | 28 | 273 | 3.996 | 2.174 |
| 13 | 124 | -0.643 | 1.214 |  | 28 | 274 | 1.718 | 1.559 |
| 13 | 125 | -0.035 | -0.485 |  | 28 | 275 | 1.145 | 2.033 |
| 13 | 126 | -0.172 | -0.266 |  | 28 | 276 | 1.172 | 2.321 |
| 13 | 127 | 0.419 | -0.718 |  | 28 | 277 | 0.992 | 1.798 |
| 13 | 128 | -0.649 | 0.176 |  | 28 | 278 | 1.796 | 2.229 |
| 13 | 129 | -0.585 | -0.800 |  | 28 | 279 | 1.792 | 2.349 |
| 13 | 130 | 0.077 | -0.679 |  | 28 | 280 | 2.661 | 1.509 |
| 14 | 131 | 0.887 | 0.747 |  | 29 | 281 | 2.515 | 2.706 |
| 14 | 132 | 0.911 | 0.448 |  | 29 | 282 | 2.084 | 2.345 |
| 14 | 133 | 2.064 | 0.082 |  | 29 | 283 | 2.779 | 2.825 |
| 14 | 134 | -0.286 | -0.209 |  | 29 | 284 | 3.393 | 3.182 |
| 14 | 135 | 2.897 | -0.097 |  | 29 | 285 | 1.313 | 2.521 |
| 14 | 136 | 1.503 | 0.761 |  | 29 | 286 | 2.140 | 2.513 |
| 14 | 137 | 1.930 | 1.347 |  | 29 | 287 | 3.394 | 2.143 |
| 14 | 138 | 1.743 | 0.951 |  | 29 | 288 | 2.550 | 2.453 |
| 14 | 139 | -0.099 | 0.848 |  | 29 | 289 | 4.865 | 2.086 |
| 14 | 140 | 0.991 | 0.570 |  | 29 | 290 | 0.381 | 2.027 |
| 15 | 141 | -1.585 | 1.588 |  | 30 | 291 | 2.026 | 1.804 |
| 15 | 142 | -0.072 | 1.169 |  | 30 | 292 | 4.510 | 3.313 |
| 15 | 143 | 2.273 | 1.255 |  | 30 | 293 | 3.941 | 3.076 |
| 15 | 144 | 0.387 | 0.926 |  | 30 | 294 | 3.741 | 3.645 |
| 15 | 145 | 2.079 | 2.127 |  | 30 | 295 | 4.131 | 3.216 |
| 15 | 146 | 2.187 | 1.315 |  | 30 | 296 | 4.040 | 3.280 |
| 15 | 147 | 1.254 | 1.046 |  | 30 | 297 | 1.542 | 2.401 |
| 15 | 148 | 3.212 | 1.787 |  | 30 | 298 | 3.934 | 3.378 |
| 15 | 149 | 2.891 | 1.164 |  | 30 | 299 | 2.418 | 2.667 |
| 15 | 150 | 0.869 | 0.847 |  | 30 | 300 | 3.023 | 3.177 |
